# Evaluation of Effectiveness of Embolization in Pelvic Congestion Syndrome with the New Vascular Occlusion Device (ArtVentive EOS™): Preliminary Results

**DOI:** 10.1007/s00270-016-1380-8

**Published:** 2016-06-01

**Authors:** Krzysztof Pyra, Sławomir Woźniak, Anna Drelich-Zbroja, Andrzej Wolski, Tomasz Jargiełło

**Affiliations:** Department of Interventional Radiology and Neuroradiology, Medical University of Lublin, Jaczewskiego 8 Street, 20-954 Lublin, Poland; III Gynecology Clinic, Medical University of Lublin, Jaczewskiego 8 Street, 20-954 Lublin, Poland

**Keywords:** Pelvic congestion syndrome, Embolization, Vascular occluder

## Abstract

**Purpose:**

This study aimed to collect confirmatory data in support of the safety and efficiency of the ArtVentive EOS™ for the treatment of the pelvic congestion syndrome (PCS). This study was based on the OCCLUDE 1 Study Protocol approved by the Local Ethics Committee.

**Materials and Methods:**

A prospective study carried out in June and July 2014 included 12 women aged 21–48 years (mean 31 years) scheduled for PCS embolization using the ArtVentive EOS™. The inclusion criteria were clinical symptoms of PCS documented by transvaginal Doppler ultrasound and pelvic MRI. The pelvic pain was assessed by VAS score from 0 to 10 (0 represents lack of pain and 10 unbearable pain). A decrease in pelvic pain intensity based on the VAS was considered a clinical success.

**Results:**

Successful embolization procedures with ArtVentive EOS™ were performed in 11 out of 12 patients. Nine patients underwent unilateral embolization of the left ovarian vein, and two had bilateral embolization of the ovarian veins. Complete ovarian vein occlusion confirmed by post deployment venography was achieved in all 11 patients. Procedures lasted from 19 to 45 min (average 28 min). Pain intensity decrease was observed in all 11 patients—a decrease of 5.6 points—from 7.3 pre-procedure to 1.6 post-embolization (standard deviation: 0.67). In one case, the left ovarian vein was injured by guide wire manipulation with contrast extravasation—not clinically significant.

**Conclusions:**

The use of ArtVentive EOS™ for occlusion of the ovarian veins in PCS patients is safe and effective.

## Introduction

Pelvic congestion syndrome (PCS) is a common cause of chronic lower abdominal/pelvic pain, estimated to affect about 10–15 % of women, predominantly between the ages of 30 and 45 [1]. PCS is anatomically characterized by the presence of varicose and incompetent parametrial veins [2]. In the majority of patients, PCS manifests with non-cyclic abdominal or pelvic pain lasting for at least half a year. Typically, it is characterized by chronic, dull and continuous discomfort, increasing on prolonged standing during menstruation and after sexual intercourse. Other symptoms include vulvar swelling, lower limb oedema and urinary urgency. Women with PCS are typically premenopausal, and a relationship between PCS and endogenous oestrogen levels has been suggested, since oestrogen is known to weaken vein walls [3–5].

1. In order to evaluate the characteristic set of clinical symptoms, imaging examinations may be useful to differentiate the causes of pain. The preferred imaging study for PCS is transvaginal ultrasound (TVUS) with Doppler imaging, which enables dynamic visualisation of the flow in pelvic venous plexus. The unaffected pelvic veins are relatively straight structures with a diameter less than 4 mm. In patients with PCS, ultrasound findings commonly include parametrial venous plexus dilation above 6 mm and slow or reversed blood flow during Valsalva manoeuvre. Important is also to find dilated arcuate veins passing through the uterine muscle. The possibility of oestrogen overstimulation in women with PCS may explain why more than 50 % also have cystic ovaries as observed during TVUS [6–8].

**Fig. 1:**
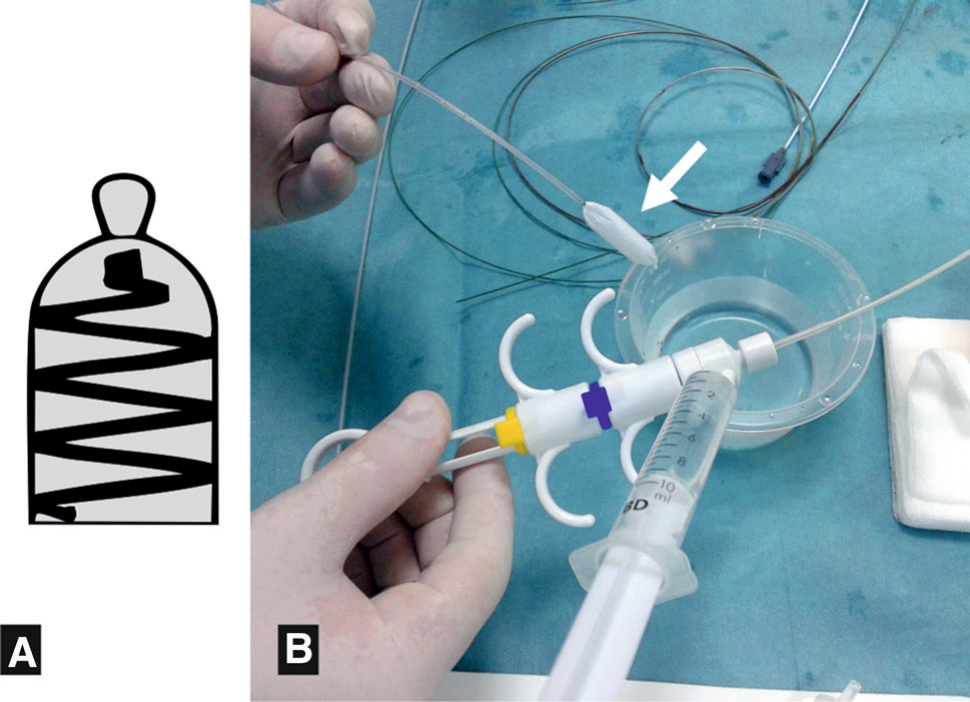
ArtVentive EOS™ schematic picture (**A**) and the occlusion system preparation (**B**)—flushing of a PTFE coating of the device (*arrow*) before placing it into introducing catheter

**Fig. 2:**
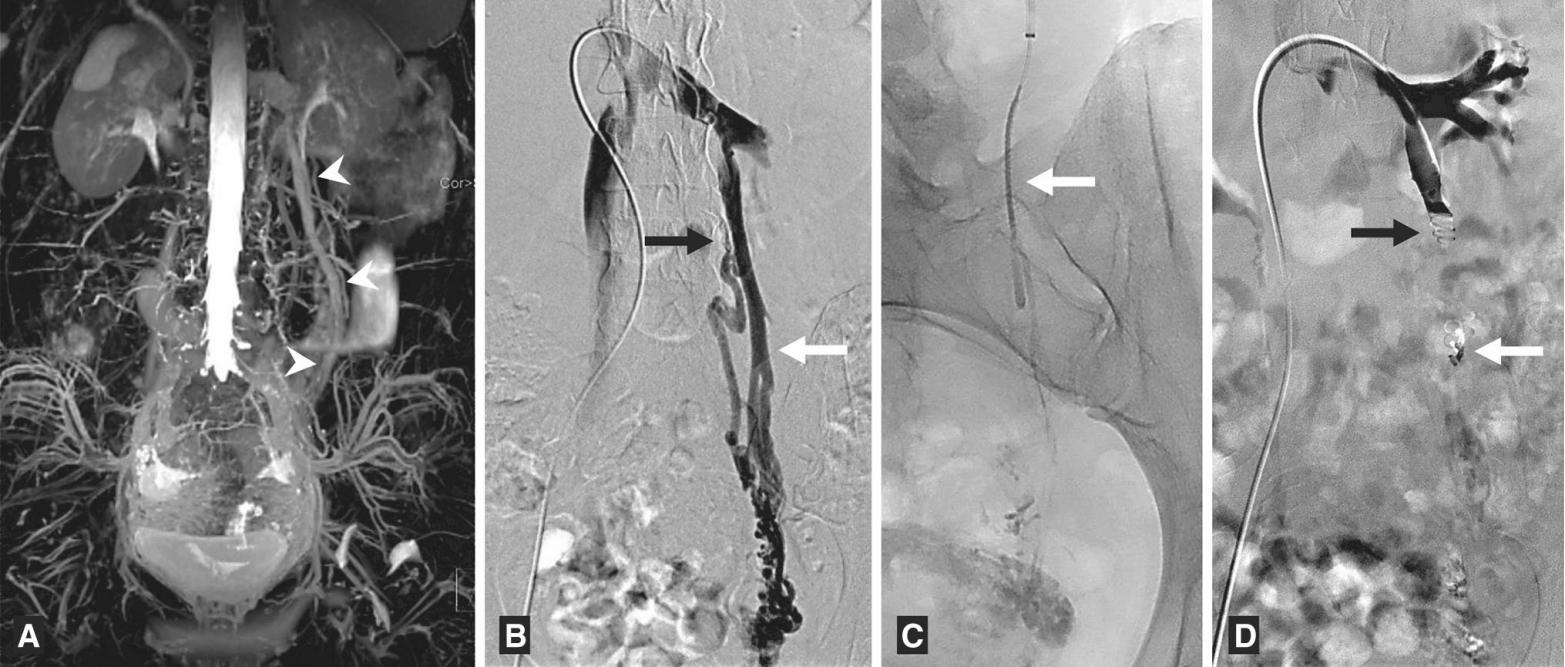
The 22-year-old female with PCS. **A** The MR appearance of the dilated left ovarian vein (*arrowheads*)—FSE T2-weighted sequence with fat-sat (MIP reconstruction). **B** Initial venography, showing a complex confluence of dilated ovarian vein. **C** The first occlusion device on position—right over the lower, major veins confluence (*white arrow*). **D** Check venography, after placement of the second occluder, implanted over the highest venous confluence (*black arrow*)

Another non-invasive imaging technique to assess the pelvic venous outflow is the MRI (Fig. 1). Using a routine FSE T2-weighted sequence with fat saturation is able to evaluate dilated ovarian veins (Fig. 2a). A volumetric single slab TSE sequence is very useful which enables acquisition of high-resolution 3D datasets (i.e. SPACE). There are also possibly more advanced techniques like time-resolved MR-angiography, which are used for not only detecting but also grading ovarian venous reflux. Unfortunately, all MR techniques are poor in assessing the possible coexisting internal iliac venous incompetence [9–11].

Endovascular diagnosis of PCS is based on venography, which remains highly effective modality. Diagnostic venography offers a direct imaging of the blood reflux into varicose plexus. It provides dynamic flow of information and accurate measurements of the ovarian and internal iliac veins [11, 12]. Venographic findings in PCS include retrograde flow in dilated ovarian veins and the presence of tortuous collateral pelvic venous pathways. Delayed clearance of injected contrast is visible [5, 13].

The treatment of PCS depends mainly on the severity of the pain. Non-steroid anti-inflammatory drugs are often used, and contraceptives have a role in cases of pain associated predominantly with menstruation [14]. Available studies also describe the use of medroxyprogesterone which alleviates the symptoms in about 40 % of patients [1, 14]. Before the advent of interventional radiology techniques (prior to the 1980s), women suffering from PCS were treated surgically with ovarian vein ligation or even subjected to total hysterectomy [15].

The first ovarian vein embolization was performed by Edwards in 1993 [16]. Since then, embolization has become the first-line treatment of PCS due to its low invasiveness and high efficacy [3–5, 17]. The findings of many prospective studies have demonstrated the efficacy up to 83 % of the procedure to be over 48 months without adverse effects, such as returned pain, menstrual disorders, disruption of the hormonal metabolism or reduced fertility [18]. Various embolization techniques have been described to occlude ovarian veins. The most often reported is the use of pushable fibered 0.035″ coils advanced through normal diagnostic catheters. The main drawback of this technique is the need to use many coils, frequently along the whole course of the vein. This is because one coil (or even few) may not give immediate, complete occlusion, especially when ovarian vein is dilated over 10 mm [5, 17, 18]. Thus, authors emphasize the need for additional administration of obliteration agents (i.e. 3 % polidocanol) [3, 4, 17]. There are also some reports on using microcatheters and platinum microcoils. Such procedures generally are considered more advanced techniques and are more expensive, thus used in more complex anatomy, when conventional techniques have failed [5, 19].

What interested us in this study was the evaluatation of the possible advantage of immediate ovarian vein occlusion using the new occlusion device (ArtVentive EOS™).

## Materials and Methods

All patients with PCS were assessed for treatment by a gynaecologist. After obtaining a medical history, each patient underwent physical examination and TVUS followed by an MRI study (FSE T2-weighted fat saturation sequences and/or 3D volumetric protocol)—showing ovarian vein dilatation and uterine plexus varicosity. None of patients in our group was diagnosed with evident coexisting vulvar venous reflux or lower limb varicosity. All the data were then evaluated by an interventional radiologist for possible embolization. The embolization procedures were performed up to the tenth day of the cycle after excluding pregnancy with a serum B-HCG test. Additional laboratory tests included platelet count, clotting time, blood urea nitrogen and creatinine.

A prospective observational study was performed in June and July 2014—twelve patients were diagnosed with PCS and scheduled for embolization. The pain intensity was assessed before and 6 months after embolization using visual analog scale (VAS)—from 0 to 10 (where 0 represents lack of pain and 10—unbearable pain). Before the procedure, the mean VAS score was 7.3 (standard deviation: 0.98). The age of patients ranged from 21 to 48 years (34 average); 1 patient was nulliparous, 4—primiparous and 7 were multiparous.

All embolization procedures were performed in the interventional radiology laboratory using a DSA system (Artis Zee, Siemens AG, Erlangen, Germany). In all cases, we used the right common femoral vein access after a local anaesthesia with 2 % lidocaine. A 7F vascular sheath was placed, and initial venography of the left renal vein was performed during Valsalva manoeuvre using a 5F Cobra II catheter. On the right side, the catheter was positioned at the right ovarian vein ostium. The ovarian veins were catheterized using a hydrophilic guidewire (Glidewire, Terumo Europe, Leuven, Belgium) and a 5F Cobra II on the left side or a 5F Simmons I on the right side (William Cook Europe, Bjaeverskov, Denmark). After successful ovarian vein catheterization, an exchange length of 260-cm Rosen exchange guidewire (William Cook Europe, Bjaeverskov, Denmark) was placed, and over this wire, a diagnostic catheter was exchanged for a guiding catheter. We used 6F and 7F deployment catheters (ArtVentive Medical, Carlsbad, CA, USA). After removing the guidewire and dilator from the deployment catheter, occluding devices were delivered to the desired place in the ovarian vein. The ArtVentive EOS™ is a spiral nitinol coil covered with an ePTFE material. It is preloaded as a double-step detachable system enabling predeployment position check, possible repositioning and final deployment. It is available in three sizes: dedicated for vessels 3.5–5, 4.5–8 and 7.5–11 mm in diameter. The first two sizes are compatible with the 6F deployment catheter and the largest one requires the 7Fr guiding catheter (Fig. 1). We used two sizes of occluders—4.5–8 mm in 4 patients and 7.5–11 mm in 7 patients. Two–five occluding devices were used in each patient—an average of two occluders per one vessel were implanted: one as distally as possible in the dilated ovarian vein trunk and the second 5–10 cm proximally. The size of each device was determined after the target vessel measurement, and it was oversized approximately by 20 % in diameter to obtain better vein occlusion and to reduce the risk of device migration.

Criteria used for unilateral (left) or bilateral embolization included clinical pain location, as well as the imaging of reverse flow through the dilated ovarian vein to the parametrial plexus—TVUS/MR findings. The final decision was made during venography with a Valsalva manoeuvre. Nine women underwent unilateral embolization of the left ovarian vein; in two cases, both right and left ovarian veins were intended to be closed with the ArtVentive EOS™—but in one patient with an extremely difficult access route (acute angulation with IVC)—the right ovarian vein was then embolized using a microcatheter (Progreat^®^, Terumo Europe, Leuven, Belgium) and microcoils (Concerto™, Covidien, Dublin, Ireland).

These procedures lasted from 19 to 45 min (average 28)—a time reflecting the total duration of the treatment from venous access to target vessel occlusion and introducer withdrawal. The mean fluoroscopy time was 13 min (ranged from 10 to 23). We also noted the radiation dose per procedure measured using a DSA system as the Reference Point Air Kerma—it was an average of 325 mGy (ranging from 146 to 1619). An average of 55 ml of contrast medium was administered per procedure.

The technical success of embolization was defined as a total occlusion of the targeted ovarian vein and no-flow of the contrast medium on check venography, during the Valsalva manoeuvre. The procedure effectiveness was evaluated 3 months after embolization—based on a history of residual pain intensity, physical examination and TVUS.

## Results

Successful embolization procedure with ArtVentive EOS™ was achieved in 11 out of 12 patients. In one patient, we could not advance the 7F deployment catheter to dilated left ovarian vein as it could not pass the proximal valve. Thus, safe occluding device implantation was not possible. The patient was then successfully treated using pushable coils implanted through the 5F diagnostic catheter which was placed distally in the ovarian vein after passing the proximal valve. In addition, in one patient, after successful left-sided ArtVentive EOS™ delivery, we were unsuccessful in placing the 6F deployment catheter in the dilated right ovarian vein. In this case, the embolization procedure was completed using a microcatheter and platinum microcoils.

**Fig. 3:**
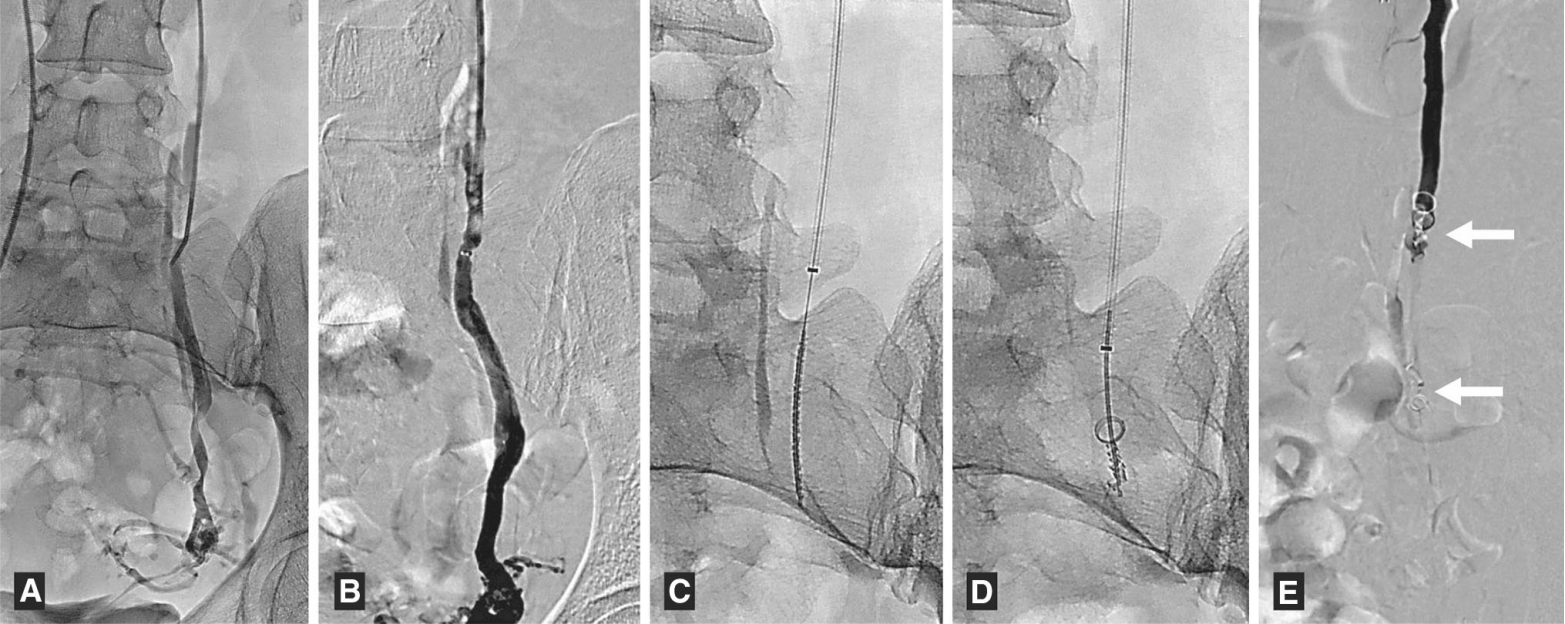
The 31-year-old female with PCS. **A** Initial venography, diagnostic catheter placed down in the main dilated ovarian vein. **B** Check venography, from introducing catheter. **C** The ArtVentive EOS™ device on position. **D** Partially deployed occluder (position correction possible). **E** The final venography after placement of the two devices—the second one over the highest confluence vein (*white arrows*)

Complete occlusion of the ovarian vein using the ArtVentive EOS™ was achieved in 11 patients (Fig. 3), including one patient embolized with the occluding device only on the left side (as described above). Eight patients were discharged from the hospital the same day as the procedure. Four women had some periprocedural discomfort and reported as prolonged pain or discomfort in the lower abdomen/pelvic region. This was resolved spontaneously before their discharge next morning.

**Fig. 4:**
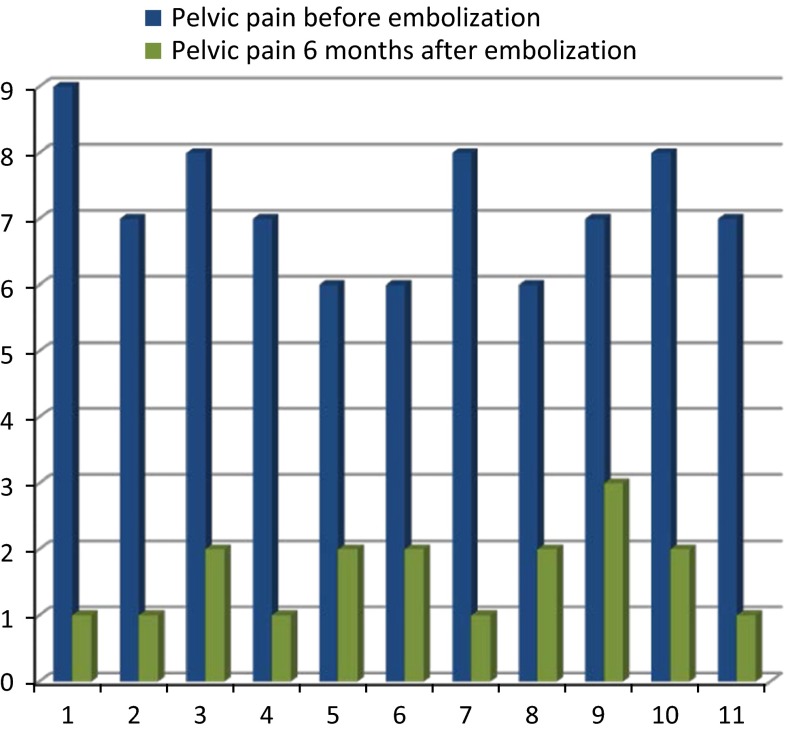
Rating VAS scale of the reported pain—before and 6 months after embolization

A reduction in pelvic pain intensity assessed using VAS was considered as clinical success and was observed in all the patients - pain decreased by 5.63 points on average (from 7.27 to 1.64 pts) in 6 months follow-up (Fig. 4). There were no major device-related complications or device malfunctions during delivery and deployment. In one case, an ovarian vein was perforated by a guidewire manipulation with the minimal contrast agent extravasation being documented. This was not clinically significant, both immediately after the procedure and during the follow-up.

## Discussion

PCS has been reported to negatively affect quality of life and personal relationships. It results in physical and psychological disorders. The latter may lead to depression and anxiety [1, 2]. Finally, pain related to PCS is frustrating both for patients and treating physicians, especially when the therapy fails [20]. It is also recognized that pelvic varicosities are often found incidentally in asymptomatic patients. According to available studies, pelvic varices occur in 38–47 % of women without PCS symptoms [1, 2, 18]. This means that only about 60 % of patients with varicose ovarian veins have symptoms of chronic pelvic pain syndrome and need treatment [1–3, 9]. Therefore, a good cooperation between gynaecologists (offering clinical assessment) and radiologists (providing appropriate imaging to direct possible intervention) is desirable for proper patients' selection for treatment.

Contemporary scientific data show that the percutaneous, endovascular ovarian vein embolization is the current standard procedure for PCS therapy with a technical effectiveness estimated at 96–100 % and recurrence incidence of 10–20 % [5, 19–21].

In the study by Laborda et al., a group of 202 patients who underwent embolization for PCS were investigated. The technical success was 100 %. In 68.3 % of patients, all 4 veins involved were closed; 3 veins were embolized in 23.8 % of patients and 2 veins in 7.9 % of patients. The mean procedural time was 43.3 ± 6.9 min. Embolization coils migrated in four cases (1.9 %). The coils were successfully removed using endovascular loop shares in three cases; in one case, the coil migrated to the lungs, and the patient refused further treatment and remained asymptomatic. No remote embolization-related complications were observed after 5 years [21].

Nasser et al. reported that a substantial decrease in pain over a 12-month period (from 7.34 to 0.47 according to VAS) was noted, despite less frequent closure of internal iliac veins, when compared to the study by Laborda et al. (LIIV 91 vs. 80 %, RIIV 74 vs. 46 %). In this group of 13 patients, four complications were observed, i.e. migration of coils, which were removed using endovascular loops. No fatal procedure-related complications were noted [22].

Although limited by the relatively small number of patients, our study showed promising results confirming the safe and effective use of the new occlusion device in the endovascular treatment of PCS. Technically, the new occluder in all successful cases, was implanted very precisely with possibility to correct its initial position. In both patients in whom we were not able to catheterize ovarian vein (one of them the left one and the second on the right site) with the deployment catheter using femoral access, it would possibly be easier to do this with jugular access. Many authors report that all the pelvic embolization procedures they perform are from jugular site. The jugular access enables easier catheterization, especially, when multi-vessel (ovarian and internal iliac veins) embolization is performed [4, 5, 17]. In patients with only ovarian veins embolization planned, the femoral access is in our opinion good enough. Some interventionists also reported good technical outcomes of glue embolization in PCS patients [4, 5, 20]. We also use cyanoacrylate glue but mainly in male varicoceles embolization, particularly when anatomy of spermatic veins is difficult and the use of microcatheter is needed. In females with PCS, the use of glue is, in our opinion (confirmed by others), limited by ovarian vein dilation exceeding 8–10 mm and higher possibility for uncontrolled liquid embolic material migration [23].

In this study, we did not use any sclerosants because we wanted to collect data of immediate, total ovarian vein occlusion without the influence of other embolic/sclerosing agents. But of course, it is possible to perform embolization with combination of occluders and other agents. Embolization performed by distal and proximal vein occlusion with the additional use of obliteration agent called “sandwich technique” is a valued technique. In every technique, the aim is to embolize the whole length of the vein. Decision concerning the selection of the most effective technique should be appropriately tailored to the specific clinical conditions.

Another observation from our study is that the use of the occluding devices may shorten procedure times and reduce radiation doses. This is very important, but it is rather an issue in PCS patients diagnosed with only ovarian veins and uterine plexus incompetence. In patients suffering from PCS with more than one or two ovarian veins affected, the use of many (more than 2–3) occluders is probably not shorter than routine coiling and is not more cost effective. The possible advantage of the use of complete occluding systems with controlled deployment is to decrease number of cases with non-target embolization (i.e. coils migrations), due to ability of repositioning. In addition, we think (according Venbrux) that immediate and total occlusion may reduce the possible necessity for retreatment [24].

Finally, we report that, the clinical results at 6 months of the use of the ArtVentive EOS™ are comparable to other studies, not only in PSC patients with dominant ovarian veins incompetence [3, 4, 18, 21, 22].

## Conclusions

The use of ArtVentive EOS™ for occlusion of the ovarian veins in PCS patients is safe and effective. All the occlusion devices were implanted as planned with good deployment control and possible correction of its initial position. During the 6-month observation, reduction of pelvic pain after ovarian veins embolization was substantial—from 7.27 to 1.64 according to VAS.
